# Vanadium

**Published:** 1831

**Authors:** 


15. *Vanadium*, a new metal found in iron ore at the mines of Fahlun. Mr. Sefstrom is its discoverer, though Bezzelius has particularly examined it. M. del Rio, of Mexico, has also found this metal in some brown lead ore, in the district of Zeinampas. He called it *Erythronium.* The name Vanadium is from an ancient deity of the Scandinavians. Vanadium combines with oxygen to form an oxyde, and also an acid, the salts of which are called *vanadates.*—*Annales de Chimie, XLV.*

